# Regional differences in historical diphtheria and scarlet fever notification rates in The Netherlands, 1905–1925: a spatial-temporal analysis

**DOI:** 10.1098/rsos.230966

**Published:** 2023-11-29

**Authors:** Scott A. McDonald, Maarten van Wijhe, Hester de Melker, Dimphey van Meijeren, Jacco Wallinga

**Affiliations:** ^1^ Centre for Infectious Disease Control, Netherlands National Institute for Public Health and the Environment, Bilthoven, The Netherlands; ^2^ PandemiX Center, Department of Science and Environment, Roskilde University, Universitetsvej 1, 4000 Roskilde, Denmark; ^3^ Department of Biomedical Data Sciences, Leiden University Medical Center, Leiden, The Netherlands

**Keywords:** scarlet fever, *Streptococcus pyogenes*, diphtheria, *Corynebacterium diphtheriae*, historical epidemiology, Netherlands

## Abstract

Background. We describe how rates of two frequently occurring notifiable diseases—diphtheria and scarlet fever—varied between regions of The Netherlands in the early twentieth century, and identify potential factors underlying this variation. Methods. Digitized weekly mandatory notification data for 1905–1925, municipality level, were aggregated into 27 ‘spatial units’ defined by unique combinations of province and population density category (high: more than 4500; mid : 1250–4500; low: less than 1250 inhabitants km^−^^2^). Generalized additive regression models were fitted to estimate the associations between notification rates and population density, infant mortality rate and household income, while adjusting for temporal trends per spatial unit. Results. Annual *per capita* notification rates for both diphtheria and scarlet fever tended to rise from the beginning of the period 1905–1925 until peaking around 1918/1919. Adjusted diphtheria notification rates were higher for high- and mid- compared with low-density municipalities (by 71.6 cases per 100 000, 95% confidence interval (CI) : 52.7–90.5; 39.0/100 k, 95% CI : 24.7–53.3, respectively). Scarlet fever showed similar associations with population density (35.7 cases per 100 000, 95% CI : 9.4–62.0; 21.4/100 k, 95% CI: 1.5–41.3). Conclusions. There was considerable spatial variation in notification rates for both diseases in early twentieth century Netherlands, which could partly be explained by factors capturing variation in living conditions and socio-economic circumstances. These findings aid understanding of contemporary respiratory infection transmission.

## 1. Introduction

Historical morbidity and mortality data are valuable for gaining understanding on the roles that improved (or worsened) socio-economic circumstances and related factors can have on population health. There is a relatively large body of research focussing on, for instance, the epidemiological transition (the change in mortality burden from infectious to chronic diseases [1]), as well as differences in mortality risk between urban and rural regions [2,3].

In The Netherlands, mortality from infectious diseases fell rapidly from about 1875 [4]; however, morbidity—as measured by the frequency of occurrence—of certain diseases exhibited an overall increasing trend; for instance, for scarlet fever such a rise is apparent for the period 1905 through to 1919 [5].

Although in modern times infectious diseases are no longer as deadly for developed countries as they were in the pre-twentieth century era, in developing countries they are responsible for a considerable disease burden, especially among infants and young children. For instance, mortality from respiratory infections among children aged 5 years or less in 2000–2019 was highest in countries in West and Central Africa [6]. The chance of acquiring an airborne infection such as influenza, diphtheria and scarlet fever in particular, is plausibly affected by socio-economic circumstances (standard of living), including factors such as access to medical treatment, nutrition, sanitation, knowledge of and adherence to hygienic practises, urban versus rural settings and population density (where crowding is conducive to transmission). There is evidence from nineteenth century Spanish data that factors such as living in a low-density (rural) setting with clean air and access to sufficient nutrition were associated with longer life expectancy [2]. Population density and poor living conditions have been proposed as explanatory factors for contemporary diphtheria outbreaks in Bangladesh [7]. We wished to explore whether similar variables could account for spatial variation in the incidence of two of the most frequently occurring notifiable diseases—diphtheria and scarlet fever—in The Netherlands in the early part of the twentieth century.

Diphtheria, a respiratory and/or cutaneous disease, is caused mainly by toxin-producing *Corynebacterium diphtheriae* that spreads though airborne transmission or by contact with wound exudate. Both forms can result in systemic disease, causing myocarditis, neuritis and cardiac death. Until the first part of the twentieth century diphtheria was a serious childhood disease, with the highest mortality rates observed for infants and young children [8]. Scarlet fever, a less deadly but frequently occurring childhood disease in the nineteenth and early twentieth century, is caused by *Streptococcus pyogenes* with symptoms including high fever, vomiting, headache, sore throat and a red rash [9].

The main objective of this study is to describe how rates of diphtheria and scarlet fever varied between regions of The Netherlands and over time in the early twentieth century, and to identify potential sources for this variation. Insights from this analysis will help understand the factors associated with transmission of historical and contemporary respiratory infections and/or with differential susceptibility to infection across regions.

## 2. Methods

Using graphical and statistical methods, we investigated temporal and spatial patterns of notified cases of diphtheria and scarlet fever over the period 1905–1925 (the years for which we had digitized data available), focussing on variation over year, population density category and geographical region.

Population density, geographical location, infant mortality rate and household income can all be seen as proxies for standard of living and so are included in our analyses as explanatory variables. For instance, high population density may indicate crowded living conditions, which are generally associated with poverty, lower air quality, worse sanitation and hygienic circumstances, and consequent greater exposure to air-, water- and food-borne disease (e.g. [4]). The level of urbanization *per se* may not be strongly predictive of morbidity or mortality. On the one hand, residents of more urbanized provinces may have been at a nutritional disadvantage compared with the rural population, for whom quality fresh food was more readily available, with more opportunity to produce it themselves [10]. On the other hand, residents of urban areas might benefit more from economic progress, substantive investment in water and sewer infrastructure, and advances in medical treatment. The third indicator, infant mortality, serves as a broad indicator of socio-economic circumstances, as lowering of mortality rates over time and reductions in regional disparities in mortality [11] are correlated with indicators of modernization and improving general living standards. Finally, household income conceivably encodes aspects of variation in socio-economic circumstances not captured by population density, geographical location and infant mortality rate.

### 2.1. Data sources

In 1865, the precursor to the Dutch Health and Youth Care Inspectorate was founded, and collection of public health statistics in the population (including infectious disease occurrences and cause-specific mortality) was initiated [12,13]. Notifiable diseases were first listed in 1872 in the so-called ‘Epidemic Law’. At this point, there were only seven diseases listed including scarlet fever and diphtheria. The 1865 law mandated physicians to report, within three days, any cases of diseases that could be a threat to public health, to the provincial inspector of health as well as to the mayor and municipal council; not complying with these rules could result in a monetary fine. As well, the mayor was legally empowered to relocate sick individuals for treatment and to take any measures deemed necessary to limit the spread of infection from affected locations.

We collected weekly notification data for diphtheria and scarlet fever from 1905 through to 1925 at municipality level from tables appearing in the weekly *Nederlands Tijdschrift voor Geneeskunde*, the principal Dutch medical journal. These tables were first digitized, and extensive data entry error checking was performed (see [5] for earlier work using these data). Data were missing for five weeks of our analysis period (i.e. 1908/week 20, 1916/week 38, 1923/weeks 19,20,32); cases for these weeks were imputed through simple interpolation between the number of cases recorded in preceding and following weeks.

Annual national-level population size was obtained from Statistics Netherlands [14]. The population increased from 5.5 million in 1905 to 7.3 million in 1925; approximately 25% of the total population lived in the four largest municipalities: Amsterdam, Rotterdam, Den Haag and Utrecht. Within our analysis period, municipality-level population size and population density was available for the years 1909, 1919 and 1925 only [15–17]; municipality population sizes for other years for the four largest cities (which exhibited the steepest growth over this period) were estimated by fitting polynomial functions to the available data (using 1909, 1919, 1925 and additionally 1899 estimates [18] for this task). Population sizes for the rest of the country for 1905–1924 were linearly extrapolated from 1925 backwards, taking into account the growth of the four largest cities and fitting to the national-level annual data.

Infant mortality rates (deaths among children less than 1-year-old per 100 live births) per municipality for the period 1914–1923 were obtained from the literature [11]. This period was deemed the most representative for our analysis period of the periods reported in this source. For the period 1914–1923, the lowest infant mortality rates were generally recorded in the provinces Friesland, Noord-Holland and Zuid-Holland, with 5.5, 5.9 and 6.6 deaths per 100 live births, respectively, while the highest mortality rates were observed in Limburg and Noord-Brabant, with 16.5 and 18.0 deaths per 100 live births, respectively [11].

Average household income at municipality level for 1924/1925 was sourced from CBS [19]. Annual income per household ranged from a low of *f* 857 in Sint Maarten to a high of *f* 6274 in Bloemendaal, both located in the province Noord-Holland.

### 2.2. Outcome definition

The notification rates for diphtheria and scarlet fever, calculated as the number of cases per 100 000 population per week and then aggregated to annual rates, were the principal outcome variables.

### 2.3. Explanatory variables

#### 2.3.1. Population density

Population density may be an influential explanatory variable for between-province differences in notification rates, as province-level density is highly skewed. We categorized municipalities according to population density as follows: (based on 1925 data from [17]): high = greater than 4500 persons km^−2^, which includes 13 municipalities; mid = 1250–4500 persons km^−2^, which includes 44 municipalities; low = less than 1250 persons km^−2^, which includes 1026 municipalities (in 1924/1925 [19]); the total number of municipalities varied slightly within our analysis period [20].

#### 2.3.2. Geographical location

Following previous work [21], municipalities were classified into three categories according to the province in which they were located: *coastal urban*: Noord-Holland, Zuid-Holland; *coastal agricultural*: Zeeland, Friesland, Groningen; and *inland rural*: Drenthe, Gelderland, Overijssel, Noord-Brabant, Utrecht and Limburg. These three categories reflect differences in factors such as transportation infrastructure and degree of market-oriented production [21]. Inland rural areas, for example, exported less food to other areas, which meant greater availability of fresh food.

#### 2.3.3. Infant mortality rates

Infant mortality is widely considered to be a useful measure of population health, as its causes are associated with determinants such as economic development, availability and quality of medical care, disease rates and general living conditions [22]. Because infant mortality rates can serve both as an indicator of the disease environment and the accessibility of healthcare, adjustment for infant mortality may therefore capture aspects of differences between urban and non-urban settings.

#### 2.3.4. Household income

Household income is a fundamental indicator of socio-economic circumstances. Compared with less affluent households, richer households would be able to afford more living space and better food, have more options to care for sick children, and generally have a higher standard of living.

### 2.4. Statistical analysis

We fitted separate generalized additive regression models (GAM) to data on diphtheria and scarlet fever to determine the associations between notification rates and the following variables: population density category, province category, infant mortality rate and household income, while adjusting for (smoothed) temporal trends (seasonality was not modelled as year was the time unit). GAM was chosen for the ease of controlling for nonlinear time trends when estimating associations with the variables of interest. Because the vast majority of municipalities reported zero notified cases in a given week, aggregation of municipalities was necessary. Thus, these data were aggregated into 27 ‘spatial units’ defined by the unique province and population density category combinations. Additive regression was carried out using the gam() function in the mgcv package [23] for R [24], with separate thinplate smoothing splines specified per spatial unit.

In a further, two-stage analysis, we first calculated the relative change in notification rates per spatial unit between 1905/1906 (the beginning of the analysis period) and 1918/1919 (the observed peak; figure 3). In the second stage, the relative change for each spatial unit was used as dependent variable in a linear regression analysis with population density category, province category, infant mortality and household income as explanatory variables. This second stage therefore estimates the associations between these variables and the relative change in notification rates between 1905/1906 and 1918/1919, with a larger coefficient corresponding to a steeper change between these time-points.

## 3. Results

**Figure 1.**
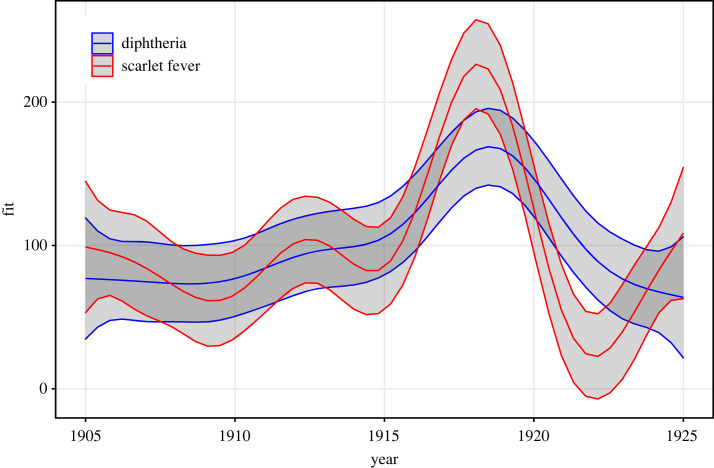
Secular trends in disease notification rates (smoothed time-series over period 1905–1925) for diphtheria and scarlet fever in The Netherlands. The *y*-axis shows predicted annual notifications per 100 000 population. Shaded areas indicate 95% confidence intervals.

Over the period 1905–1925, a total of 117 900 cases of diphtheria and 148 300 cases of scarlet fever were reported in The Netherlands. Figure 1 shows the smoothed secular trends in notification rates for both diseases, and the smoothed seasonal effect is provided in the electronic supplementary material, figure S1. Notification rates for both diseases tended to rise from the beginning of our analysis period, in 1905/1906 (65 and 108 cases per 100 000 population for diphtheria and scarlet fever, respectively) until peaking around 1918/1919, at 184 and 256 per 100 000 for the two respective diseases, followed by a decline. Both diseases exhibited seasonal patterns (electronic supplementary material, figure S1), with troughs in notification rates in late spring (scarlet fever) or late summer (diphtheria).

**Figure 2.**
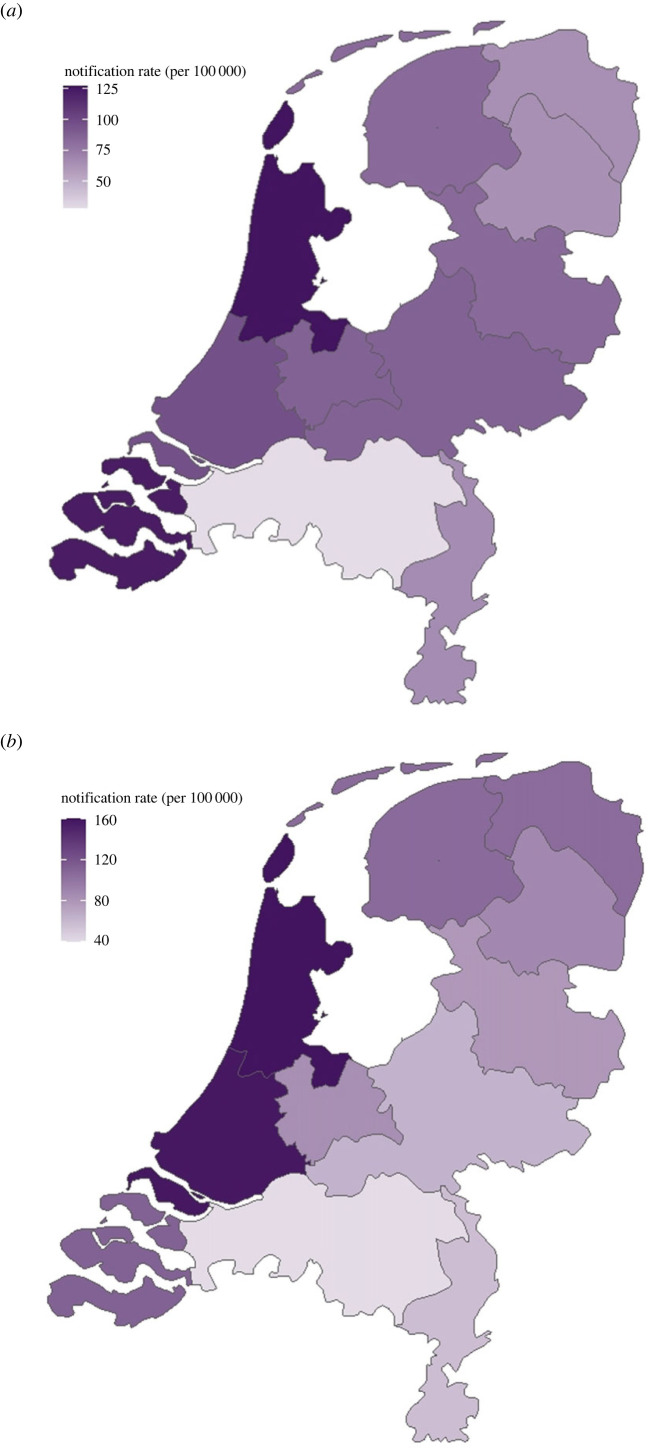
Spatial heterogeneity (at province level) in aggregated notification rates, where the aggregated notification rates per 100 000 population is calculated from the total reported cases over the entire analysis period (1905–1925); shown for (*a*) diphtheria and (*b*) scarlet fever.

**Figure 3.**
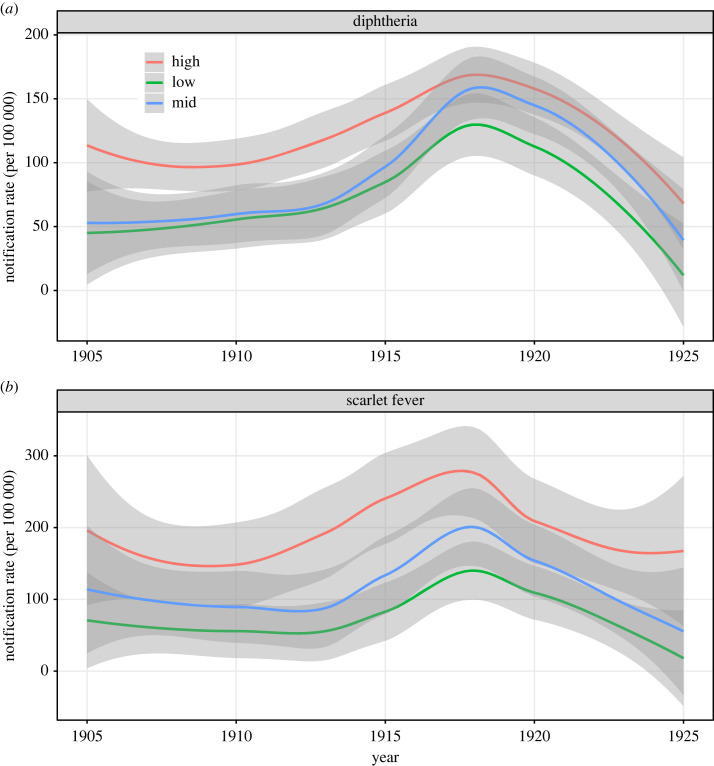
Smoothed annual notification rates (per 100 000 population) stratified by population density category over the period 1905–1925, for (*a*) diphtheria and (*b*) scarlet fever. Shading indicates 95% confidence intervals.

Considering total notified cases over the analysis period, rates were highest for both diseases in the province Noord-Holland, followed by Zeeland (diphtheria) and Zuid-Holland (scarlet fever) (figure 2). Stratification of national-level annual aggregated rates by population density category indicated a pattern of association between notification rates and population density (figure 3): rates were generally highest in high-density municipalities and lowest in low-density municipalities. Further stratification by province category indicated that notification rates—although data are noisy—within each population density category varied by province category (electronic supplementary material, figure S2). For diphtheria only, the single high-density municipality (Harlingen in Friesland province) within the *coastal agricultural* provinces exhibited clear outbreaks in 1913 and 1917 that were not present in other regions.

Infant mortality varied by population density category. Infant mortality rates in 1914–1923 were highest for the low-density municipalities (8.7 deaths per 100 live births, s.d. of rates in individual municipalities = 3.2), lowest for the high-density category (6.0 deaths per 100 live births, s.d. = 1.7), with the mid-density category falling in-between (8.1 deaths per 100 live births, s.d. = 3.0) [11]. Across municipalities, income was weakly negatively correlated with infant mortality (*r* = −0.28).

Results of regression analyses of the dataset organized by spatial unit are shown in table 1 and the electronic supplementary material, figures S3 and S4. For diphtheria, adjusting for other covariates, notification rates were higher (by 71.6 cases per 100 000; 95% confidence interval (CI): 52.7–90.5) for high- compared with low-density municipalities. Rates in mid-population density municipalities were also higher than in low-density municipalities (39.0 cases per 100 000; 95% CI: 24.7–53.3), but there were no significant differences according to broad geographical location after adjustment for the other variables.

**Table 1.** Regression coefficients for four covariates from univariable and multivariable generalized additive models fitted to notification rates for each province/density category combination (spatial unit), with separate per-unit spline fits to calendar year. (For the categorical covariates, coefficients (*B*) indicate the difference in annual notifications per 100 000 population compared with the reference category.)

	diphtheria		scarlet fever	
	unadj. *B* (95% CI)	adjusted *B* (95% CI)	unadj. *B* (95% CI)	adjusted *B* (95% CI)
population density category				
low (*m* = 1026)	ref.	ref.	ref.	ref.
mid (*m* = 44)	13.8 (2.5, 25.0)	39.0 (24.7, 53.3)	34.6 (19.5, 49.8)	21.4 (1.5, 41.3)
high (*m* = 13)	47.0 (33.9, 60.1)	71.6 (52.7, 90.5)	62.7 (45.1, 80.3)	35.7 (9.4, 62.0)
province category^a^				
inland rural (*m* = 567)	ref.	ref.	ref.	ref.
coastal agricultural (*m* = 208)	33.3 (21.2, 45.3)	6.3 (−7.5, 20.1)	30.6 (14.5, 46.7)	25.9 (6.7, 45.0)
coastal urban (*m* = 308)	28.8 (16.0, 41.5)	7.8 (−5.8, 21.3)	58.3 (41.4, 75.3)	41.0 (22.1, 59.8)
infant mortality rate (deaths per 100 live births)	−10.4 (−12.5, −8.2)	−8.4 (−11.2, −5.5)	−10.9 (−13.9, −7.9)	−3.4 (−7.4, 0.5)
annual household income (per *f* 100)	2.3 (0.3, 4.3)	−8.4 (−11.4, −5.3)	9.1 (6.6, 11.7)	3.3 (−0.9, 7.5)

^a^*Inland rural*: Drenthe, Gelderland, Overijssel, Noord-Brabant, Utrecht, Limburg. *Coastal agricultural*: Zeeland, Friesland, Groningen. *Coastal urban*: Noord-Holland, Zuid-Holland. *m* refers to the number of municipalities in each category, based on the total number (*n* = 1083) of municipalities in 1924/1925 [19].

For scarlet fever, after adjustment notification rates were also higher for high-density (by 35.7 cases per 100 000; 95% CI: 9.4–62.0) and for mid-density (by 21.4 cases per 100 000; 95% CI: 1.5–41.3) compared with low-density municipalities (table 1). Both *coastal agricultural* and *coastal urban* provinces were associated with significantly higher notification rates than *inland rural* provinces (by 25.9 and 41.0 per 100 000, respectively).

For both diseases, a negative relationship with infant mortality rate was observed; after adjustment for other factors notification rates decreased by 8.4 (diphtheria) and 3.4 (scarlet fever; not statistically significant) per 100 000 population for every unit increase in infant mortality (i.e. deaths per 100 live births). Household income exhibited a statistical significant positive relationship with notification rate for diphtheria only; after adjustment, rates decreased by 8.4 per 100 000 population for every 100 guilder increase in annual income. By contrast, scarlet fever notification rates increased by 3.3 per 100 guilder increase, but this was not statistically significant.

The electronic supplementary material, figure S5 shows the relative change in disease notification rates between 1905/1906 and 1918/1919 per province. Considering province/density category units, the relative change in notification rates between 1905/1906 and 1918/1919 varied from a small decrease (0.91) in Limburg (low-density category, for scarlet fever) to a very large 39-fold increase in Friesland (high-density category, consisting of a single town, Harlingen, for scarlet fever). Histograms of log-transformed relative change per disease are shown in the electronic supplementary material, figure S6. Regression models fitted to the log-transformed relative change in notification rate, with density category, province category, infant mortality rate and income specified as covariates, indicated that compared with *inland rural* provinces, *coastal urban* provinces were significantly associated with a less steep increase in the diphtheria notification rate (*B* = −0.86, 95% CI: −1.61, −0.10), but this was not observed for scarlet fever.

## 4. Discussion

Our analysis investigated spatial and temporal variation in historical notification rates for diphtheria and scarlet fever during the period 1905–1925 in The Netherlands. For both infections, incidence increased around the start of the First World War and reached a peak around 1918–1919, followed by a decline. After adjusting for secular trends and the other investigated variables, municipalities with high population density (compared with low density) were significantly associated with higher disease notification rates. For scarlet fever only, *coastal agricultural* and *coastal urban* (compared with *inland rural*) were also associated with higher notification rates. In as much as population density and urbanization reflect variation in living circumstances, these results support a relationship between the general concept of standard of living and the spread of infectious diseases and/or the susceptibility to infection. Analysis of contemporary outbreaks of diphtheria, such as in Bangladesh in 2017–2019, identified the same drivers—high population density and poor living conditions [7]—that are suggested by our historical analysis.

Despite the lower average household wealth and more restricted access to medical care among rural residents relative to urban residents, the uncrowded housing conditions, better sanitation and availability of fresh food (and consequent better nutrition) afforded by less urbanized living circumstances may explain this pattern of findings. Results are consistent with empirical historical mortality studies (e.g. [25]) and with dynamic models of infectious disease transmission in which roles for density-dependence (via the effective contact rate) on the rate of transmission are fundamental [26,27].

The small, but statistically significant, negative relationship observed between diphtheria notification rates and infant mortality rates (across spatial units) seems counterintuitive. Household income had the expected relationship with diphtheria notification rates, with higher income being associated with lower rates (across spatial units). Consistent with this result, a proxy for household income, wealth tax, was shown by Wolleswinkel-van den Bosch *et al*. [4] to be associated with the rate of mortality decline around the turn of the twentieth century in The Netherlands. It is possible that income and its correlates are important factors for the prevention of premature mortality among adults as well as infants, but other factors that are associated with rural living are probably more relevant for transmission of—and potentially, though less likely, their susceptibility to—infection. The lower overall infant mortality rates for the high-density municipalities, despite generally higher notification rates in these municipalities, might be explained in differences in susceptible population size (i.e. lower infant mortality means relatively more susceptible living children aged older than 1 year, all else being equal) or points to an unmeasured additional factor [28]. In addition, the geographical regions used in this analysis, while already of a high resolution, may nevertheless hide underlying local associations with our covariates [29,30]. For example, while overall population density is highest in urban regions, people living in rural parts probably also live close together in small communities or as families sharing a farmhouse. Similar highly heterogeneous local variation may also be present for household income and infant mortality.

Although increased notification rates between 1905/1906 and 1918/1919 were observed for both diphtheria and scarlet fever and in 26 of 27 spatial units, the rate of increase was not associated with any of the explored covariates, except that a less steep increase for diphtheria was observed for municipalities in *coastal agricultural* provinces. Given that notifications increased in virtually all spatial units (the sole exception being the low-density municipalities in the inland rural province Limburg), we assume that an underlying pervasive increase in transmission is the main factor for the rise in notification rates. By the start of our analysis period, all provinces of The Netherlands were reasonably well-connected by railway, road or waterway. Other time-varying drivers of transmission include mobilization and large-scale people movements such as the influx of Belgian refugees and prisoner internship during the First World War [31]. Wartime food shortages and the 1918/1919 influenza pandemic may also have led to increasing susceptibility to infection until about 1919. We note that during the Second World War, in which large-scale people movements also occurred, a large increase in the number of diphtheria notifications was observed in The Netherlands and in other European countries [32,33]. The proposed time-varying factors affecting transmission are supported by data on mortality from childhood infections, as temporal patterns were very similar, though mortality rates declined overall [34,35].

Contemporary diphtheria outbreaks mainly occur in regions that have low vaccination coverage and/or are affected by humanitarian crises [36]. Introduction of mass childhood vaccination against diphtheria in the 1950s in The Netherlands drastically reduced the number of diphtheria cases in the following decades [37], minimizing all other (potential) risk factors for a diphtheria infection. In the first two decades of the twenty-first century, only 18 diphtheria cases (range of 0–4 cases per year) were reported in The Netherlands. These cases mainly presented with cutaneous diphtheria, against which vaccination has low effectiveness [38]. Human-to-human transmission of *C. diphtheriae* was not recorded, and almost all *C. diphtheriae* cases (8 out of 9) had acquired infection in African or southeast Asian countries. Of the diphtheria cases acquired in the Netherlands, most (9 out of 10) were infected by *Corynebacterium ulcerans*, a zoonotic species that does not transmit between humans and can be as pathogenic as *C. diphtheriae,* which has been notifiable in The Netherlands since 2009 [39]. *Corynebacterium ulcerans* may have been responsible for a number of the observed cases in the early twentieth century, but this cannot be proved based on available data.

We note the following limitations. First, because this is an ecological analysis, one cannot attribute causality to the investigated factors. Second, we lacked information regarding under-reporting; for example, if reporting rates were lower in rural compared with urbanized regions, variation in true notified case incidence may be attenuated. Reporting rates may also have been subject to error because of misdiagnosis, for which information is scarce. Diagnoses were based solely on clinical symptoms, which in many cases could not clearly be attributed to a particular disease. Scarlet fever was confused with other rash-causing diseases such as measles, and diphtheria was difficult to distinguish from other causes of tonsillitis. One report from London for 1900–1904 indicated that scarlet fever was misdiagnosed in 4.7–6.7% of hospital-admitted fever cases, and diphtheria was misdiagnosed 8.3–16.1% of the time [40]. How these estimates might translate to community cases in The Netherlands is unknown. Because there is very limited data on the degree of misdiagnosis and under-reporting, it is unclear how either factor may have affected our analysis, though impact is probably limited*.* Third, the classification of municipalities to population density category was according to 1925 density data, and this classification was assumed to be constant over time. Fourth, potential spatial or temporal dependency in notifications was not accounted for in the statistical analysis, which will not affect point estimates but may have resulted in overly narrow 95% CIs. Finally, collinearity was a potential issue for the multivariable regression analyses—density category and province category are correlated, as seen by attenuation of regression coefficients in the multivariable analysis—however, inclusion of both factors was justified from previous research and they nevertheless are distinguishable (electronic supplementary material, figure S2).

In conclusion, there was substantial spatial variation in notification rates for diphtheria and scarlet fever in The Netherlands at the beginning of the twentieth century, which could be explained—in part—by variation in municipality population density, broad geographical location (coastal versus inland), household income, and infant mortality rate. Spatial variation in the extent that notification rates increased between 1905 and 1918 was not adequately explained by any of the investigated factors.

## Data Availability

Scarlet fever and diphtheria notification data for the analysis period are supplied as the electronic supplementary material [41].
